# A First Y-Chromosomal Haplotype Network to Investigate Male-Driven Population Dynamics in Domestic and Wild Bactrian Camels

**DOI:** 10.3389/fgene.2019.00423

**Published:** 2019-05-21

**Authors:** Sabine Felkel, Barbara Wallner, Battsesteg Chuluunbat, Adiya Yadamsuren, Bernard Faye, Gottfried Brem, Chris Walzer, Pamela A. Burger

**Affiliations:** ^1^Institute of Animal Breeding and Genetics, University of Veterinary Medicine Vienna, Vienna, Austria; ^2^Vienna Graduate School of Population Genetics, Vienna, Austria; ^3^Laboratory of Genetics, Institute of Biology, Mongolian Academy of Sciences, Ulaanbaatar, Mongolia; ^4^Institute of Remote Sensing and Digital Earth, Chinese Academy of Sciences, Beijing, China; ^5^Wild Camel Protection Foundation Mongolia, Ulaanbaatar, Mongolia; ^6^CIRAD-ES, UMR 112, Campus International de Baillarguet, Montpellier, France; ^7^Research Institute of Wildlife Ecology, Department of Integrative Biology and Evolution, Vetmeduni Vienna, Vienna, Austria; ^8^Wildlife Conservation Society, Wildlife Health Program, Bronx, NY, United States

**Keywords:** old world camelids, paternal lineage, Y chromosome, haplotype, diversity, conservation

## Abstract

Polymorphic markers on the male-specific part of the Y chromosome (MSY) provide useful information for tracking male genealogies. While maternal lineages are well studied in Old World camelids using mitochondrial DNA, the lack of a Y-chromosomal reference sequence hampers the analysis of male-driven demographics. Recently, a shotgun assembly of the horse MSY was generated based on short read next generation sequencing data. The haplotype network resulting from single copy MSY variants using the assembly as a reference revealed sufficient resolution to trace individual male lines in this species. In a similar approach we generated a 3.8 Mbp sized assembly of the MSY of *Camelus bactrianus.* The camel MSY assembly was used as a reference for variant calling using short read data from eight Old World camelid individuals. Based on 596 single nucleotide variants we revealed a Y-phylogenetic network with seven haplotypes. Wild and domestic Bactrian camels were clearly separated into two different haplogroups with an estimated divergence time of 26,999 ± 2,268 years. Unexpectedly, one wild camel clustered into the domestic Bactrian camels’ haplogroup. The observation of a domestic paternal lineage within the wild camel population is concerning in view of the importance to conserve the genetic integrity of these highly endangered species in their natural habitat.

## Introduction

The male-specific region (MSY) of the mammalian Y chromosome is transferred directly from the father to the son without recombination. This unique mode of inheritance highlights the MSY as an ideal marker to study male genealogies alongside and in comparison to maternal phylogenies based on the mitochondrial DNA (mtDNA). Due to the lack of recombination, allelic states of single nucleotide variants (SNVs) on the MSY can be combined into haplotypes and robust MSY haplotype phylogenies can be built using the principle of maximum parsimony (reviewed in Jobling and Tyler-Smith, 2017). Such MSY backbone phylogenies based on biallelic markers (Skaletsky et al., 2003) became recently available in dogs (Oetjens et al., 2018), cattle (Chen H. et al., 2018) and horses (Wallner et al., 2017; Felkel et al., 2018). The MSY revealed new insights into the domestication and uncovered male mediated historic radiations in these species. However, the Y chromosome is often not considered in genome assemblies because of the challenges to assemble its repetitive content. Hence, re-sequencing approaches to study paternal lineages often need to start with assembly work. We and others have recently shown the value that *de novo* assemblies of the MSY, generated from short read next generation sequencing (NGS) data, had for tracing male lineages (Wallner et al., 2017). We also described in detail the need to accurately define single copy regions in the assembly (Felkel et al., 2019) to unambiguously call variants in these regions only. With this approach (*de novo* assembly and SNV calling in single copy MSY regions) we elevated the resolution and accuracy of paternal lineage tracing in horses to a level similar to that in humans (Wallner et al., 2017; Felkel et al., 2019), where MSY haplotype (HT) trajectories are well described (Jobling and Tyler-Smith, 2017).

In Old World camels, domestication and historic demography have been investigated mainly by mtDNA. Domestication of dromedaries (*Camelus dromedarius*) took first place at the east coast of the Arabian Peninsula around 3,000 to 4,000 years ago (ya) (Almathen et al., 2016). Bactrian camels were domesticated around 4,000 to 6,000 ya. The described long-term divergence of 1.5 to two million years between wild (*Camelus ferus*) and domestic (*Camelus bactrianus*) two-humped camel mtDNA lineages (Mohandesan et al., 2017) predates the timeframe of camel domestication by far. Over the last century, the critically endangered wild two-humped camels (Hare, 2008) have been reduced to only ∼2,000 individuals restricted to the Mongolian Great Gobi strictly protected area “A” (GGSPAA) (Yadamsuren et al., 2012) and to the Chinese deserts Taklamakan and Lop Noor (Lei et al., 2012), where they are stringently protected. However, habitat decline, poaching, environmental pollution due to illegal mining and hybridisation with domestic Bactrian camels continue threatening the wild camels. Severely reduced mtDNA haplotype diversity (Silbermayr et al., 2010; Mohandesan et al., 2017) and low genome-wide nucleotide diversity (Fitak et al., 2016b) have been observed in the wild populations.

To trace the paternal lineages and reconstruct male-driven population dynamics in Old World camels, specifically in the highly endangered wild camel, we adopted the approach recently described in horses (Wallner et al., 2017; Felkel et al., 2019) and *de novo* assembled the Bactrian camel MSY from short read NGS data. The assembly was used as a reference for mapping and single copy MSY variant calling using short read data from five wild and three domestic Bactrian camels one dromedary as outgroup. With this, we created a first backbone Y phylogeny for Old World camels based on slowly evolving biallelic markers.

## Materials and Methods

### Data Origin

Trimmed Illumina short reads from nine wild (five male/four female) and five domestic Bactrian camel (three male/two female) genomes were derived from an ongoing project (FWF P24706-B25; PI: PB) to detect signatures of selection related to domestication in Old World camelids. In addition, one male dromedary was included as outgroup. Sample details are provided in Supplementary Table S1 and in the ethics statement below.

### MSY *de novo* Assembly

**FIGURE 1:**
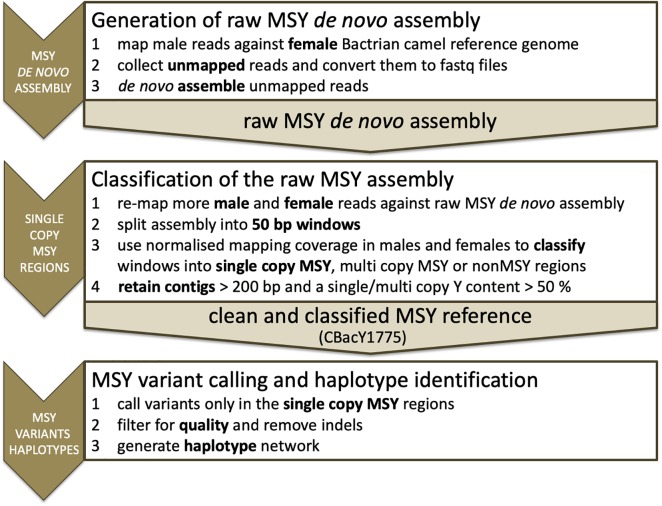
Schematic overview of the analytic steps.

Short reads of the deepest sequenced domestic male (sample ID DC269 in Supplementary Table S1) were mapped to the female Bactrian camel reference genome (JARL01; SAMN02053968; Jirimutu et al., 2012) using bwa aln (Li and Durbin, 2009). Unmapped read pairs were collected and converted to fastq files with samtools (Li et al., 2009), bedtools (Quinlan and Hall, 2010) and Unix commands. The resulting read pairs were *de novo* assembled with SPAdes (Bankevich et al., 2012). A schematic overview of the analytic steps is presented in Figure 1. For a detailed bioinformatic protocol see Supplementary Methods.

### Classification of Single Copy MSY Regions

We mapped the Illumina short reads from the eight male and six female Bactrian camels to the raw assembly output using bwa aln, removed PCR duplicates and filtered for mapping quality > 20 using samtools. We next splitted the assembly into 50 bp windows and followed the probabilistic approach of Felkel et al. (2019), which uses the differences of normalized mapping coverages in males and females to classify the windows into single copy (scY) and multi copy (mcY) MSY and not MSY (nonMSY). For the classification we did not consider DC269 on which the reference is based. For details see Supplementary Methods and Supplementary Figures S1–S5. Contigs shorter than 200 bp and with a scY/mcY-specific content less than 50% were discarded, resulting in clean and classified MSY reference contigs.

### MSY Variant Calling, Haplotype Identification and SNV Validation

Variant calling with GenomeAnalysisTK HaplotypeCaller and CombineGVCFs was performed using the mappings from above. Only variants called in scY regions (Supplementary Table S2), were used for downstream analyses. We excluded reference errors, phased variants, insertions and deletions (indels) and variants with multiple alternatives, heterozygous or empty calls from further analyses. A read depth of at least three in one individual and a genotype quality higher nine were set as limit to keep the variant in the list. The resulting variants were used to generate a haplotype network with Network (Bandelt et al., 1999). Missing variants (in low coverage samples or due to ambiguous mappings) have been implemented according to the samples’ clustering in the network. Finally, we performed independent validation of nine SNVs via Sanger sequencing. Validation candidates and primer information are shown in Supplementary Table S3. Further details are given in Supplementary Methods.

### Diversity Estimates of the Bactrian Camel MSY

**FIGURE 2:**
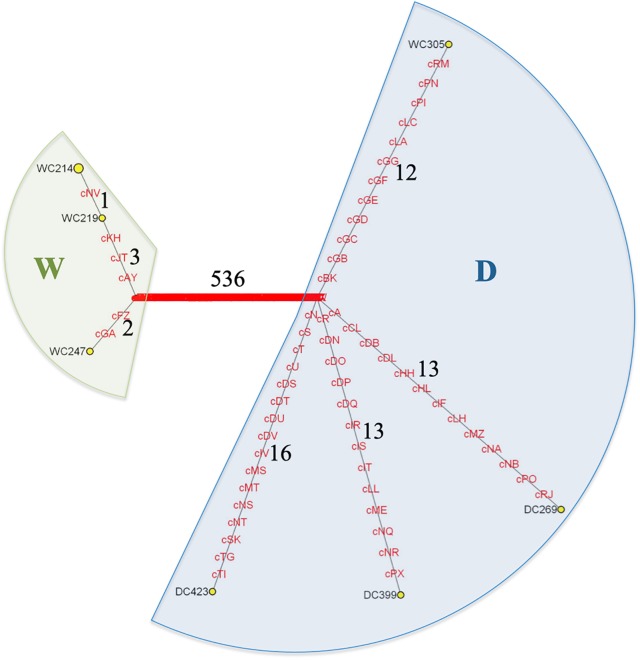
MSY haplotype network based on 596 scY SNVs found on CBacY1775 for three domestic (DC) and five wild (WC) Bactrian camels. The outgroup is not shown. HG W (green shade) is clearly separated from HG D (blue shade). Each sample has a private haplotype except for WC214 and WC218 (circle size of WC214 corresponds to both these samples comprising this certain haplotype), which share the same father (Hare, pers. comm.). WC305 is the only wild camel belonging to HG D. Numbers indicate branch lengths (numbers of mutations). The variants correspond to those in Supplementary Table S3.

Nucleotide diversity (π and 𝜃) was calculated in R for different scenarios using the whole set of SNVs detected in all individuals but the outgroup (numbers shown in Figure 2).

### Divergence Time Estimates Between Wild and Domestic Bactrian Camel Male Lineages

Felkel et al. (2019) calculated a mutation rate of 1.68 × 10^-8^ mutations/site/generation for the horse Y based on deep pedigrees and this rate is highly similar to the genome-wide estimate in humans (Kong et al., 2012). Since no pedigrees have been available to calculate a camel specific mutation rate, we used the horse rate to date nodes in the resulting camel Y network using rho statistics implemented in Network (for details see Supplementary Methods; variants and contigs used for dating are indicated in Supplementary Tables S3, S4, respectively; the haplotype network based on variants used for dating is shown in Supplementary Figure S6).

## Results

### Bactrian Camel MSY *de novo* Assembly

We used 11,987,466 read pairs not mapping to the female Bactrian camel reference genome as input for the *de novo* assembly and classified the resulting 22,398 contigs of the raw assembly into scY, mcY, and nonMSY regions. An overview of the assembly and classification statistics is given in Table 1. We kept contigs longer than 200 bp and with a Y-specific content > 50% (Supplementary Figure S5) and retrieved the final MSY assembly (CBacY1775) with a total size of 3.8 Mbp distributed over 1,775 contigs. CBacY1775 has a minimum/maximum length of 201/87,065 bp and an N50 of 858 bp. We classified 2.39 Mbp as scY, 1.21 Mbp as mcY and 0.18 Mbp as nonMSY, respectively. scY and mcY regions are provided in Supplementary Table S2 and the assembly can be downloaded from NCBI (RYZT00000000). Contig lengths are provided in Supplementary Table S4. Mappings are uploaded to the NCBI public database (Accession numbers are provided in Supplementary Table S1).

**Table 1:** *De novo* assembly and classification statistics of the Bactrian camel MSY.

	Total length [Mbp]	Number of contigs	N50 [bp]	scY [Mbp]	mcY [Mbp]	nonY [Mbp]
Raw assembly	14.49	22,398	308	2.47	1.50	10.52
CBacY1775	3.79	1,775	878	2.39	1.21	0.18


### MSY Variation in Wild and Domestic Bactrian Camels to Build a Phylogenetic Network

For MSY haplotype reconstructions we considered only variants called in scY regions of three domestic and five wild two-humped camels. Based on a total of 596 variants (Supplementary Table S3) we distinguished seven haplotypes that formed two clearly separated haplogroups (HG), W and D. When displayed in a phylogenetic network (Figure 2), HG W was private for the wild camels in our dataset, whereas HG D was detected in three domestic individuals plus one wild individual (WC305). Within HG D, the domestic Bactrian camels formed three branches, each of them with a length of 13–16 SNVs. The wild camel WC305, which unexpectedly clustered into the principally domestic HG D, had a branch length similar to the three domestic lineages (twelve SNVs). The wild camels in HG W had only two lineages with two to four variants from the basal node of the HG. Based on all detected variants we obtained diversity estimates given in Table 2.

**Table 2:** MSY diversity estimates in domestic and wild Bactrian camels.

	𝜃	π
All domestic Bactrian camels	1.17^∗^10^-5^	1.17^∗^10^-5^
Wild Bactrian camels without WC305	1.37^∗^10^-6^	1.67^∗^10^-6^
All wild Bactrian camels	1.11^∗^10^-4^	1.73^∗^10^-4^


We verified nine randomly selected SNV using PCR and Sanger sequencing. Six SNVs showed the expected MSY specific amplification pattern and a PCR amplicon was revealed only from male template DNA, whereas no product was generated when using female Bactrian camel DNA as template. The SNVs in these six loci were confirmed with Sanger sequencing (Supplementary Table S3, Supplementary Methods, and Supplementary Figure S7). However, three primer pairs developed for SNV loci (Supplementary Table S3) also amplified DNA in females. Although showing a single band on an agarose gel in males and females, these products could be sequenced only for one SNV (cGA). The other two SNVs could not be validated as the interferences observed in the Sanger sequence electropherograms indicated a cross amplification of MSY, autosomal and/or X-chromosomal regions. Alternative target-specific primers need to be developed for these two loci to amplify the MSY region unambiguously. Such cross amplifications are not unexpected, keeping in mind the genomic structure of the MSY (Jobling and Tyler-Smith, 2003; Skinner et al., 2013) with varying, often substantial sequence homology to X and autosomal regions.

### Estimation of the Divergence Time Between Wild and Domestic Bactrian Camel Paternal Lineages

Using the horse Y mutation rate previously obtained and assuming a generation time of 6 years in Bactrian camels, the most recent common ancestor (MRCA) of haplogroups D and W was estimated to 26,999 ± 2,268 years before present (ybp; Supplementary Figure S6). The MRCAs of HG D and W were estimated to 1,764 ± 416 ybp and 490 ± 353 ybp, respectively.

## Discussion

### *De novo* Assembly of the MSY Region in Bactrian Camels

In this study, we generated a 3.8 Mbp sized *de novo* assembly of the MSY region of domestic Bactrian camels and classified scY regions following the very conservative approach by Felkel et al. (2019). We then created a first Y phylogeny of highly endangered wild Bactrian camels and their domestic congeners, using a dromedary as outgroup. The size of the newly assembled MSY in Bactrian camels (3.8 Mbp) is smaller than that of horses (6.58 Mbp; Felkel et al., 2019). Apart from one male *Camelus ferus* shotgun sequenced genome (Jirimutu et al., 2012), currently available camel reference genomes were generated from female data (Wu et al., 2014; Fitak et al., 2016a; Elbers et al., 2019), which makes them not useful for improving the assembly. Thus, long read sequencing (e.g., PacBio or Nanopore) will be necessary to retrieve a more complete MSY assembly of Old World camels. Future comparisons with the alpaca MSY (Jevit et al., 2018) will be very informative to shed light on the paternal side of the evolutionary history in Old and New World camelids.

We successfully used CBacY1775 as reference to map shotgun reads from three domestic and five wild Bactrian camels and to ascertain scY SNVs (Supplementary Table S3). Based on 596 variants we distinguished seven haplotypes that separated into two haplogroups (W and D). Recently, Chen N. et al. (2018) investigated 29 Y-chromosomal sequence tags and 40 bovine-derived microsatellites in 94 Chinese domestic Bactrian camels. They detected the same Y-chromosomal haplotype in all tested sequence tags but one, which showed an indel, and observed allelic variation at only one microsatellite (USP9Y-STR) leading to three different HTs. This highlights the importance for further joint efforts to examine camel Y diversity.

### Low MSY Sequence Variation Within Wild Bactrian Camels

The diversity on the MSY in wild two-humped camels (π = 1.67^∗^10^-6^, without the D haplotype carrier WC305) seems to be much reduced compared to that of domestic Bactrian camels (π = 1.17^∗^10^-5^). This may reflect the decline of the wild camel population in Mongolia over the past decades (Hare, 2008; Yadamsuren et al., 2012). The higher diversity observed in domestic compared to wild camels seems reasonable given that less than ∼2,000 wild two-humped camels are still living in Mongolia and China (Yadamsuren et al., 2012), contrary to more than 20,000 domestic Bactrian camels (FAO, 2017). Future analyses of the MSY in more wild Bactrian camels are necessary to monitor and understand the consequences of the low paternal diversity in these endangered animals, also in view of inbreeding and a successful conservation management.

### Male-Driven Population Dynamics in Domestic and Wild Bactrian Camels

Based on 596 SNVs (Supplementary Table S3) we created a Y-phylogenetic network and observed a clear separation of wild and domestic Bactrian camels into two different haplogroups (W and D). This finding is consistent with the separation observed in mtDNA based genealogies (Silbermayr et al., 2010; Mohandesan et al., 2017). The only exception turned out to be the wild Bactrian camel WC305, which clustered with the domestic camels into haplogroup D (Figure 2). This male wild camel was captured during a radio-collaring mission in the GGSPAA, where officially no domestic Bactrian camels are allowed to roam. Notably, WC305 had a wild camel mtDNA haplotype (Mohandesan et al., 2017), but we found a signature of introgressed domestic alleles in this wild camel individual on the autosomal genome (Fitak et al., 2016b). So far, our MSY observation in WC305 could be interpreted a footprint of a domestic male that escaped into the wild. This inference is based on the assumption that the lineages of the populations ancestral to wild and domestic camels had differentiated before domestication. Alternatively, under the scenario of incomplete lineage sorting, the existence of wild camels carrying the D lineage would not be unexpected. The next necessary step will be haplotyping of a large number of male wild and domestic Bactrian camels to determine MSY haplotype frequencies. Such data should enlighten the male-driven population dynamics in these two sister species in more detail. Additional autosomal markers could also provide information on the magnitude of the hybridisation between domestic and wild Bactrian camels, which is favored by some Mongolian camel owners due to the effect of hybrid vigor (Silbermayr and Burger, 2012; Yadamsuren et al., 2012).

### Divergence Time Estimates of Paternal and Maternal Genealogies in Wild and Domestic Bactrian Camels Are Not Consistent

The mitochondrial genomes of wild and domestic Bactrian camels coalesce 1.1 (0.58–1.8) mya (Mohandesan et al., 2017). This is much older than the divergence time estimate of 26,999 ± 2,268 ya based on the MSY. Inconsistencies between maternal and paternal estimates of the time to the MRCA have been described in horses (Wallner et al., 2017) and might be caused by a faster mutation rate in the mtDNA, the maintenance of older maternal lineages compared to the paternal counterpart and/or possibly be a signature of sex-specific generation times. The estimated time of the MRCA (1,764 ( 416 ya) of the domestic Bactrian haplogroup suggests that all MSY lineages in our dataset derive from a single ancestor that lived after the species domestication around 4,000- to 6,000 ya (Peters and von den Driesch, 1997). The very recent approximated MRCA (490 ± 353 ya) of the wild camels reflects a strong bottleneck in males following the dramatic population decline of these highly endangered animals over the last century.

### Conservation of Male Lineages in Wild Bactrian Camels

Until now, no large-scale study has been performed to identify male genealogies in the last remaining and highly endangered wild two-humped camels in the Mongolian GGSPAA and in the Chinese deserts Lop Noor and Taklamakan. A comprehensive survey including a large number of male individuals will be necessary in the future to establish a more comprehensive view on the diversity, introgression patterns and divergence estimates within and between wild and domestic Bactrian camels. Here, we provide the basis for developing a SNV array for a continuous screening of paternal lineages to investigate male-driven population dynamics in Old World camels. This will contribute to conserve the integrity of the wild two-humped camel gene pool.

## Ethics Statement

EDTA blood samples for domestic and wild Bactrian camels were retrieved commensally during routine veterinary controls, micro-chipping or radio-collaring of Mongolian wild camels, respectively. All data sets were collected within the frames of the legal requirements of Austria and Mongolia. Micro-chipping of semi-wild camels from the breeding center of the Wild Camel Protection Foundation (WCPF) in Mongolia was performed with the request and consent of the WCPF. Capture and collaring of wild camels within the Mongolian GGSPAA (Walzer et al., 2012) was conducted within a cooperation agreement between the International Takhi Group and the Mongolian Ministry of Nature, Environment and Tourism signed on 15.02.2001 and renewed on 27.01.2011.

## Author Contributions

SF performed bioinformatic analyses and lab work for variant validation. SF and BW developed the methodology and wrote and discussed the manuscript. BC, AY, BF, and CW provided samples and interpreted results. GB provided resources and discussions. PB conceived and managed the project and wrote the manuscript. All authors edited the manuscript.

## Conflict of Interest Statement

The authors declare that the research was conducted in the absence of any commercial or financial relationships that could be construed as a potential conflict of interest.
