# Supplementary figures and images for: A First Y-Chromosomal Haplotype Network to Investigate Male-Driven Population Dynamics in Domestic and Wild Bactrian Camels
Source: Front Genet. 2019 May 21;10:423. doi: 10.3389/fgene.2019.00423 (PMC6537670; doi:10.3389/fgene.2019.00423)

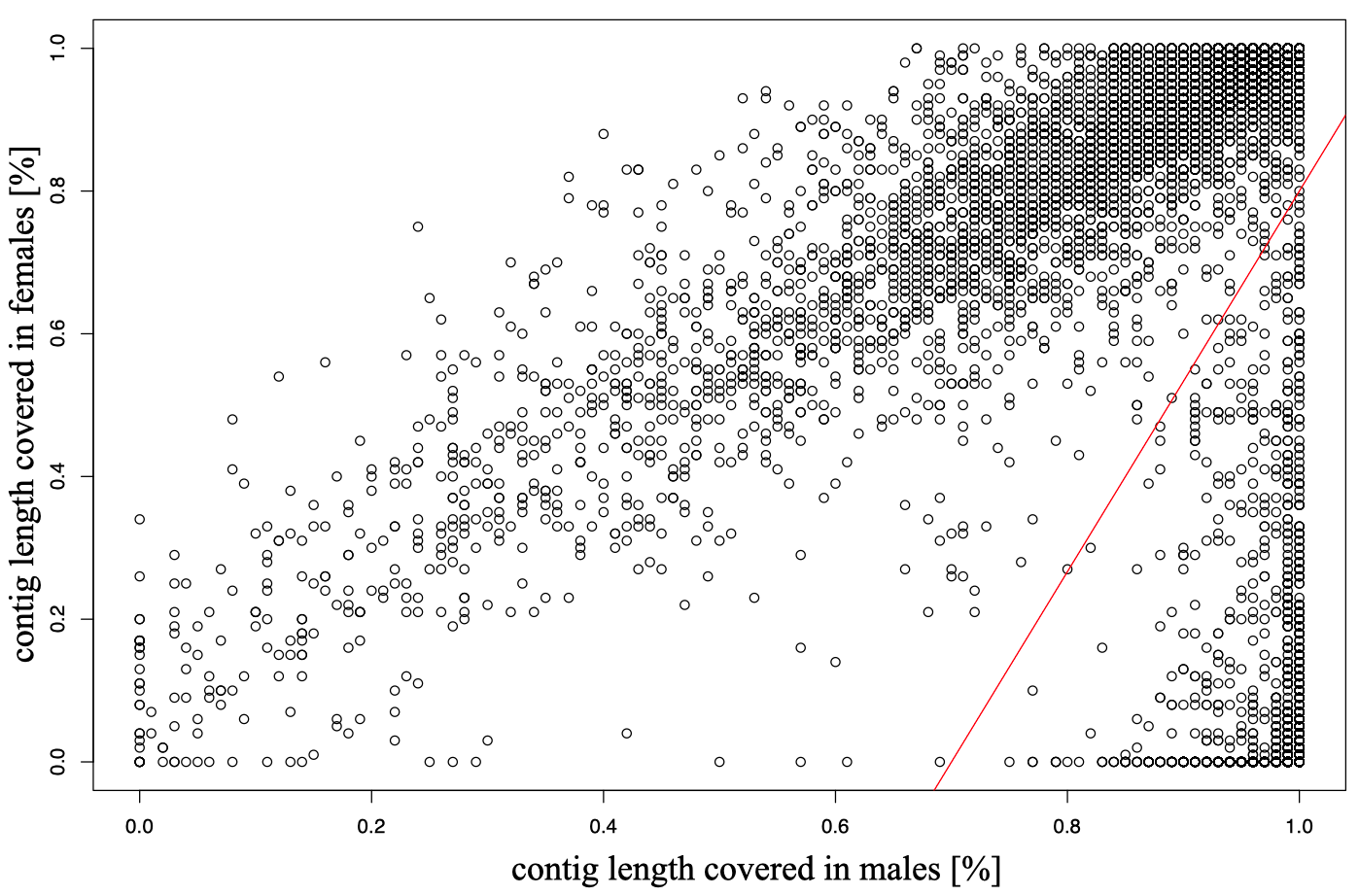

Supplement: Supplementary file 5 [file Image_1.jpeg]

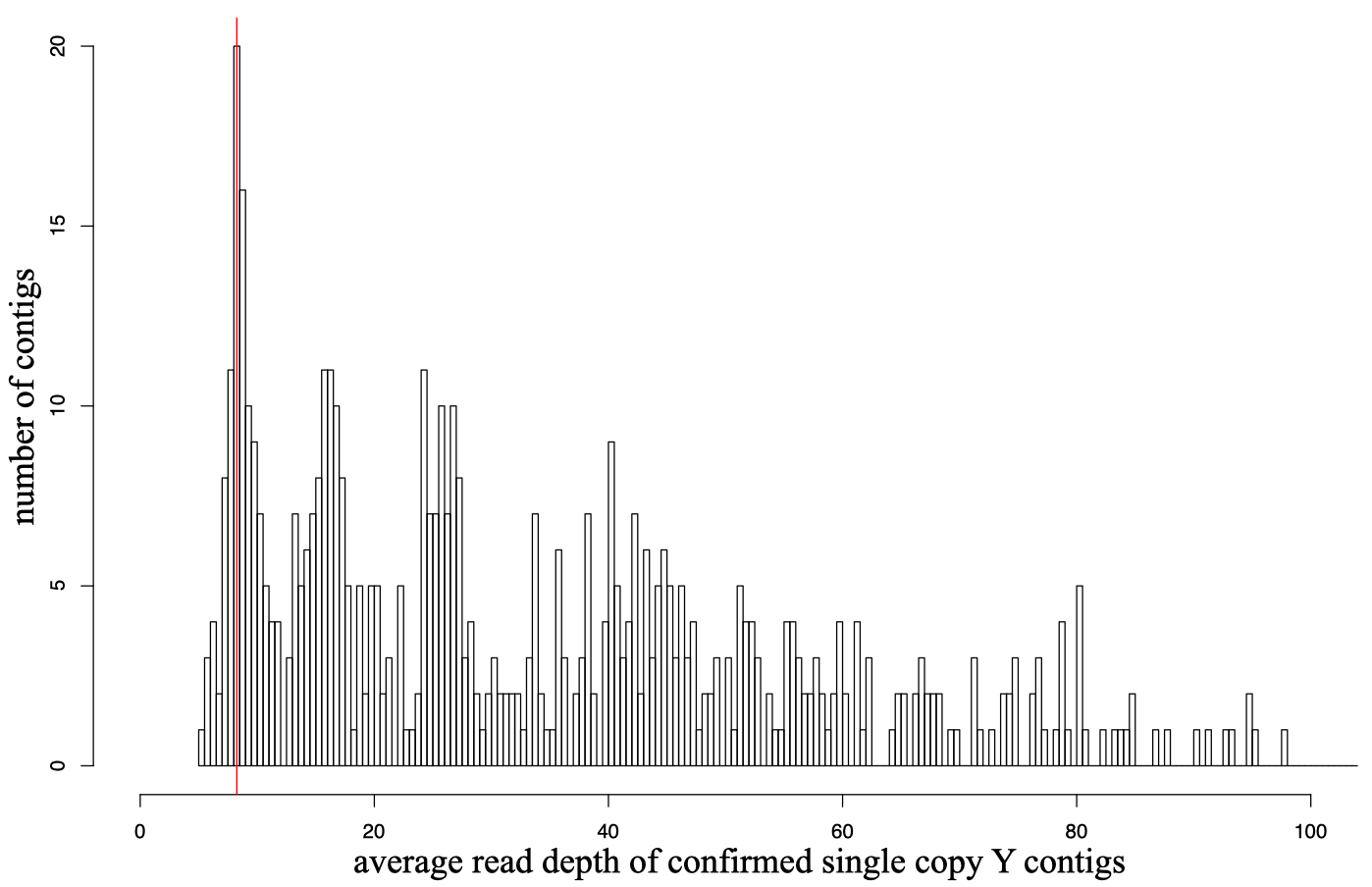

Supplement: Supplementary file 6 [file Image_2.jpeg]

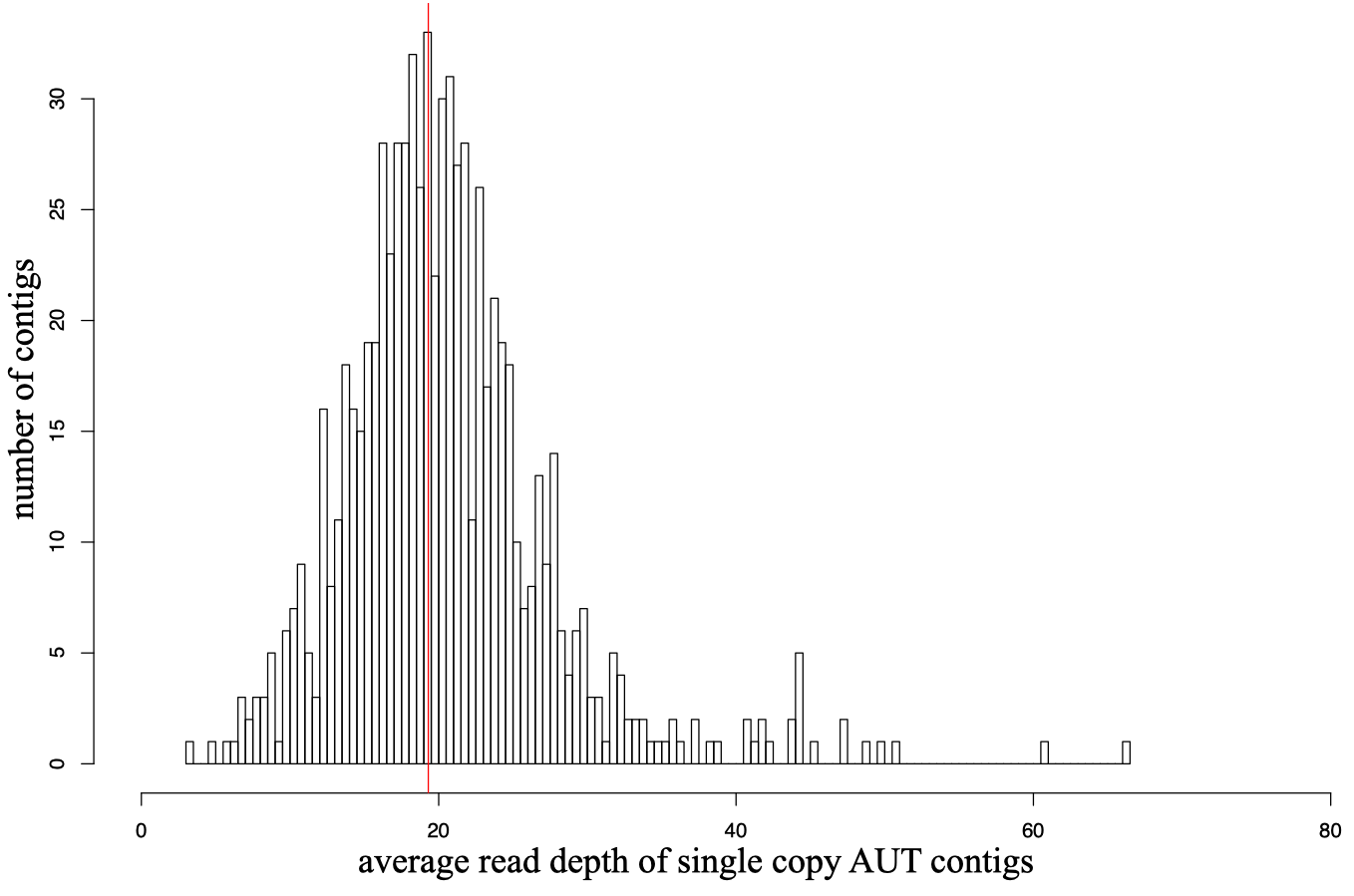

Supplement: Supplementary file 7 [file Image_3.jpeg]

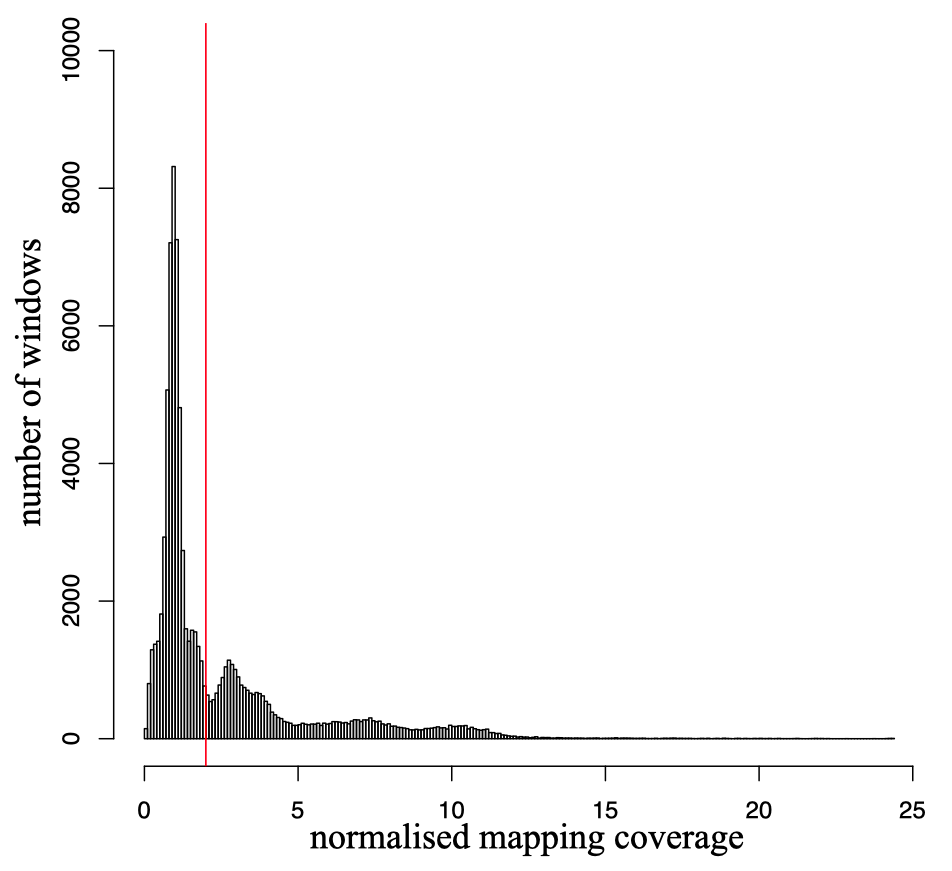

Supplement: Supplementary file 8 [file Image_4.jpeg]

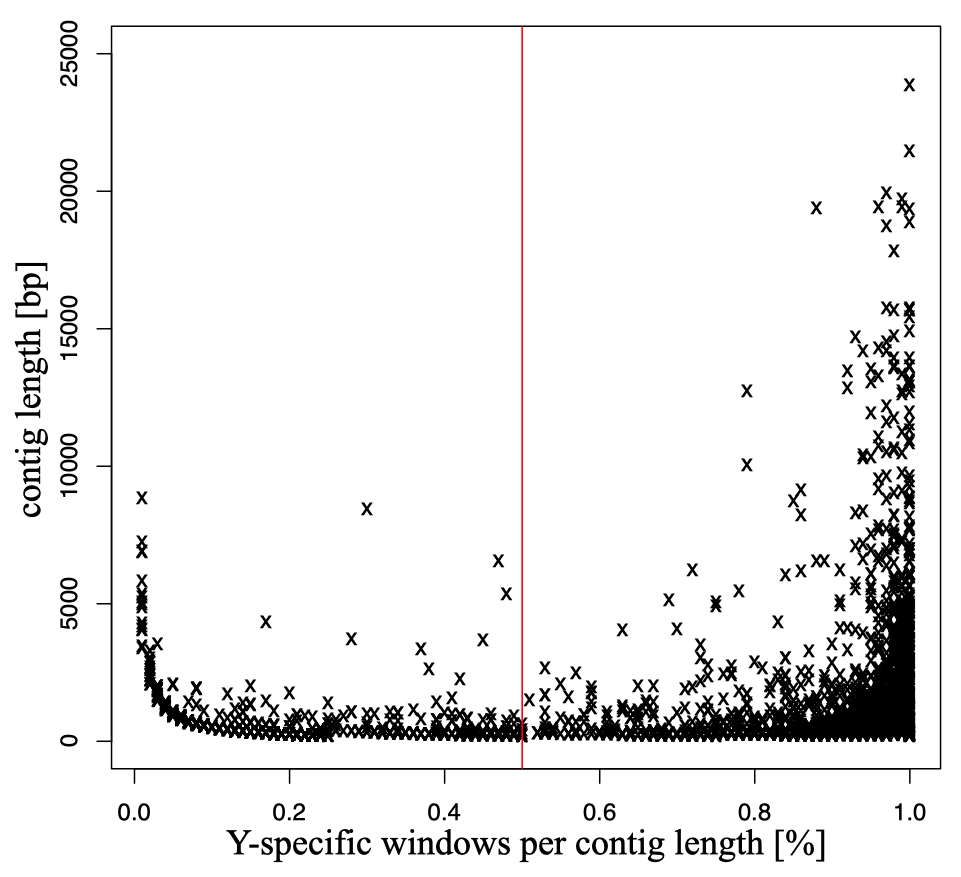

Supplement: Supplementary file 9 [file Image_5.jpeg]

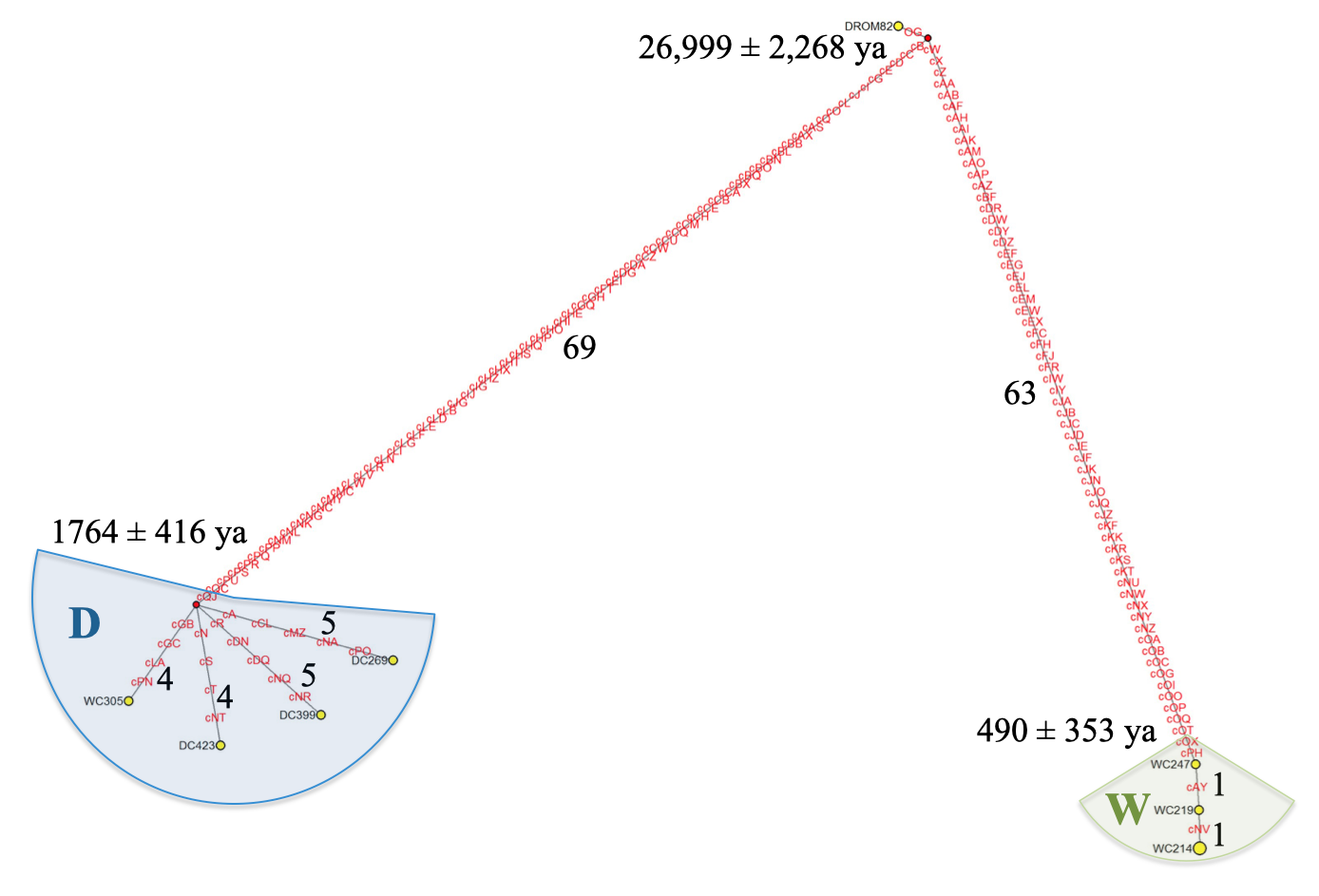

Supplement: Supplementary file 10 [file Image_6.jpeg]

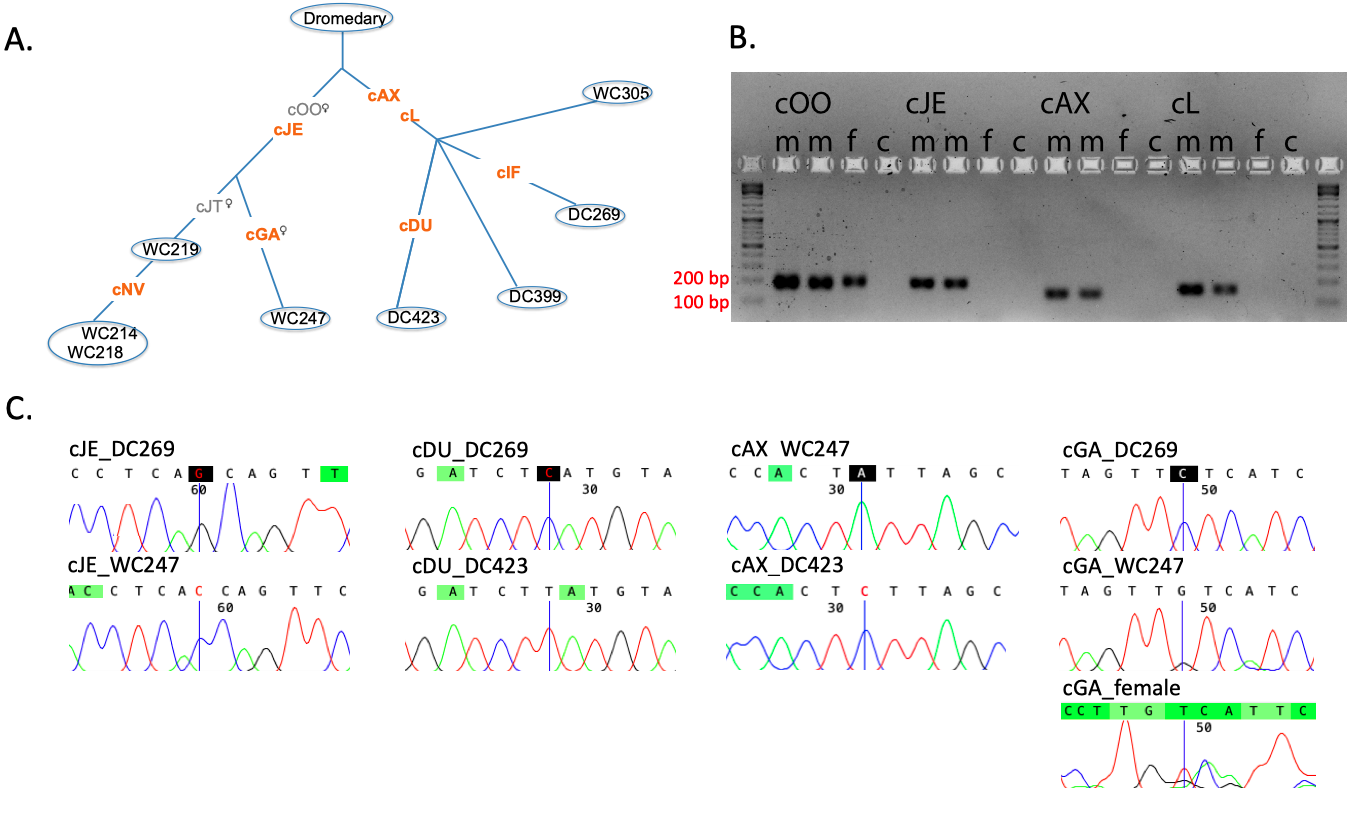

Supplement: Supplementary file 11 [file Image_7.jpeg]
