# Supplementary material for: A First Y-Chromosomal Haplotype Network to Investigate Male-Driven Population Dynamics in Domestic and Wild Bactrian Camels
Source: Front Genet. 2019 May 21;10:423. doi: 10.3389/fgene.2019.00423 (PMC6537670; doi:10.3389/fgene.2019.00423)
Supplement: Supplementary file 12 [file Data_Sheet_1.docx]

**Supplementary Methods**

## *Samples and raw data processing* Removing of adaptor sequences and quality-based trimming was performed with ReadTools v0.2.1 TrimFastq.

## *Generation of the raw Y-chromosomal assembly Y read enrichment*

We first mapped the whole-genome Illumina sequenced reads from the Bactrian camel sample DC269 to the female Bactrian camel reference genome JARL0 using bwa aln v0.7.15 (-t 8 -n 0.02 -l 200). We removed duplicates, extracted only unmapped read pairs and converted them to fastq format with samtools v1.4 and Python v3.5.1.

*Assembly*

A *de novo* assembly was generated with SPAdes v3.11.1 using the read pairs obtained in the previous step.

*Classification of Y-specific single copy and multi copy regions to obtain CBacY1775*

*Mapping*

We mapped all eight males and six females and the male dromedary to the raw assembly with bwa aln v0.7.15 (-t 8 -n 0.02 -l 200), removed duplicates and filtered for mapped reads with a mapping quality of > 20 using samtools v1.4. The raw assembly was then separated into 50 bp windows to run the classification pipeline according to Felkel et al. (2019). Unix commands and bedtools v2.25.0 makewindows were used.

*a) Mapping coverage normalisation*

As sequencing-depth differs among individuals (see Supplementary Table S1), we calculated and normalised each window’s mean coverage using bedtools, Python and R scripts. A table with the observed mean coverages per window (row) and individual (column) was provided in the final classification R script shown below. In the original approach (Felkel et al., 2019) mean autosomal single copy window coverages were inferred based on mappings to the pseudoautosomal region of the X-chromosome (PAR) and used to normalise the data such that a relative coverage of one corresponds to a diploid state. However, since the PAR is not yet described on the X-chromosomal reference of *Camelus bactrianus* it was necessary to modify the normalisation: First, for each contig we plotted the percentage of its length covered on average by males and females to identify pure Y-specific contigs (100% covered by males and 0% by females, see Supplementary Figure S1). Second, per male, we generated a histogram of these contigs’ mean coverages and took the mode of the first peak (single copy peak; see Supplementary Figure S2) in the distribution as the mean single copy Y coverage to get the denominator for the normalisation. Third, based on the resulting male denominators we were able to define autosomal single copy contigs (above line in Supplementary Figure S1). Contigs defined as autosomal single copy in all males were used to infer the denominators (half of the mean autosomal single copy coverages) for the normalisation of females by also, per female, plotting a histogram of their read depths and taking the mode of the first peak (single copy peak; see Supplementary Figure S3) in the distribution as the mean autosomal single copy coverage. For each camel, these modes were calculated in R v3.2.3 and used in the final classification R script to normalise the Y assembly mean window coverages such that a relative coverage of one corresponds to a haploid (or hemizygous, with respect to the Y chromosome) state.

*b) Calculation of female background coverage in confirmed Y regions*

It is necessary to allow for spuriously mapped reads of females to run the model from Felkel et al. (2019). Per female, this background coverage was estimated by calculating the mean window coverage for pure and putative Y-specific contigs (below line in Supplementary Figure S1). These values were provided in the final classification R script.

*c) Probabilistic model - calculation*

Apart from the normalisation and background coverage calculation we sticked to the protocol described in Felkel et al. (2019; the final classification R script is shown below) to determine single copy Y regions. The R script is based on the following formula derived by Felkel et al. (2019): The normalised coverages in 50 bp windows were modelled as Poisson distributed. Each Y assembly window was assigned to one of two classes: MSY or nonMSY. The observed mean coverages per window (*k*) and camel (*i* for males and *j* for females) to get the normalised mapping coverages (*y*), each camels’s denominator for the normalisation (*c*) and the female background coverages (*b*), all obtained as described above, are provided as input for the R script (table “malfem”, vector “mal_par” and vector “fem_par” and vector “constants”). Assuming equal prior probabilities, the probability of assignment of a window to a class can be calculated from this ratio (for details and derivation of the formula see Felkel et al., 2019):


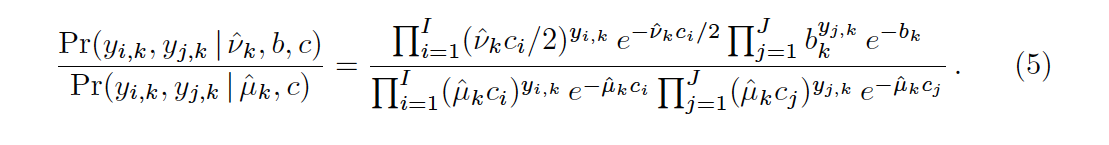


Calculations have been performed with R v3.2.3.

# final classification R script:

malfem=read.table("males+females_win50_meancov.txt")

mal_par <- c(provide meansinglecopyYwindowcoverages:male1,male2,(…),maleX)

fem_par <- c(provide meansinglecopyAUTwindowcoverages/2):female1,female2,(…),femaleX)

constants <- c(provide meanpureandputativeYwindowcoverages:female1,female2,(…),femaleX)

nwindows=length(malfem[,1])

start=1

nwindows

estimated_1_m=estimated_mf5=estimated_mf5_m1f2=rep(-1,nwindows)

total_likeli1=total_likeli2=total_likeli3=rep(0.5,nwindows)

for(i in start:nwindows){

#define the columns with male data 🡪 4 to 10 in our case

mal=as.numeric(malfem[i,4:10])

#define the columns with female data 🡪 11 to 16 in our case

fem=as.numeric(malfem[i,11:16])

malfem=c(mal,fem)

malfem_par=c(mal_par,fem_par)

estimated_1_m[i]=m1=sum(mal)/sum(mal_par)

estimated_mf5[i]=m2=sum(malfem)/sum(malfem_par)

if((m1>0) & (m2>0)){

#here you calculate the window’s likelihood for being MSY and nonMSY:

total_likeli1[i]=sum(mal*log(m1*mal_par)-m1*mal_par)+sum(fem*log(constants5)-constants5)

total_likeli2[i]=sum(malfem*log(m2*malfem_par)-m2*malfem_par)

}

}

#now extract windows that have higher likelihood to be MSY:

prob1=1/(exp(total_likeli2 - total_likeli1)+1)

idx=prob1>0.5

hist(estimated_1_m[idx],xlim=c(0,25),ylim=c(0,10000),nclass=250,main=paste("histogram of Y-specific windows"),xlab="relative copynumber",ylab="number of windows")

#threshold 2 was chosen based on hist above, can be adjusted

idx1=prob1>0.5 & estimated_1_m<2

idx2=prob1>0.5 & estimated_1_m >=2

#save single-copy and multi-copy MSY windows as separate lists

window_id=data.frame(malfem[,1][idx1],malfem[,2][idx1],malfem[,3][idx1])

write.table(window_id,file="Y_Assembly_scY_windows.txt")

window_id=data.frame(malfem[,1][idx2],malfem[,2][idx2],malfem[,3][idx2])

write.table(window_id,file="Y_Assembly_mcY_windows.txt")

A cutoff of two was chosen to separate scY and mcY windows (Supplementary Figure S4). In the final step we excluded contigs with less than 50% of their length classified as scY or mcY (Supplementary Figure S5). The result is the final CBacY1775 assembly (RYZT00000000) and the classified windows are given in Supplementary Table S2.

## *Variant calling and haplotype ascertainment*

We used the mappings from above and performed variant calling using GenomeAnalysisTK v3.7 HaplotypeCaller and CombineGVCFs. We used the methods described in Felkel et al. (2019) for variant filtering to generate a first Y genealogy for the two-humped Old-World camels: First, only variants found in scY windows were considered and then phased variants, variants with multiple alternatives and reference errors were excluded. In the third step a read depth of at least three in one individual and a genotype quality higher nine were set as limit to keep the variant in the list. In the last step, variants with heterozygous or no-calls in at least one of the samples were excluded. Additionally, we excluded indels. The haplotype network was visualised with Network v4.6.15.

*Validation of variants by Sanger sequencing*

We randomly picked nine variants distributed all over the haplotype network and validated them by Sanger sequencing. Primers were designed using the webtool Primer3 v0.4.0 (variants and primers are shown in Supplementary Table S2 and Figure S7). PCR was performed in a 20 µl volume containing 2µl genomic DNA (5 – 20 ng/µl), 0.5 µM of each primer, 1.5 mM MgCl and 1 x PCR buffer, 200 µM each dNTP and 0,1 U Taq DNA polymerase (Agrobiogen). The DNA was initially denatured at 95°C for 5 min, followed by 35 cycles of 30 s at 95°C, 30 s at annealing temperature (Supplementary Table S2) and 40 s at 72°C. After the 35 cycles a final extension for 4 min at 72°C was performed. PCR products were visualised on a 2% agarose gel and purified using QIAquick PCR purification kit. Concentration of the products were checked on a 2% agarose gel using DNA ladder and then sent for Sanger sequencing to LGC genomics®.

*Dating*

For dating we considered only variants lying on contigs covered to at least 99% by all males plus the outgroup individual to assure correct positioning of the root (see Supplementary Table S4 and Supplementary Figure S6). In 329 contigs (0.90 Mbp; 904,006 bp scY) covered to at least 99% by all male samples incl. the outgroup we found 154 variants (of which two are excluded for dating because they have a third variant in the dromedary: cGZ and cGJ). We dated the most important nodes using rho statistics implemented in Network and assuming the horse Y mutation rate of 1.6916*10^-8^ mutations/site/generation from Felkel et al. (2019). We assumed a generation time of six years for camels, such that we obtained a mutation rate of 2.8193*10^-9^ mutations/site/year.

*Accessions*

Bamfiles (Accessions in Supplementary Table S1, Bioproject PRJNA510735) and the final reference CBacY1775 (RYZT00000000) plus classification results (Supplementary Table S2) are provided. The variants are provided in Supplementary Table S3. If the bioinformatic pipeline provided in the Supplementary Methods is not sufficient, a full bioinformatic code is available upon request from the corresponding author.

*List of tools used*

| **Tool** | **Reference/Link** |
| --- | --- |
| **bedtools v2.25.0** | **Quinlan, A. R. & Hall, I. M. BEDTools : a flexible suite of utilities for comparing genomic features. Bioinformatics 26, 841–842 (2010).** |
| **bwa v0.7.15-r1140** | **Li, H. & Durbin, R. Fast and accurate short read alignment with Burrows – Wheeler transform. Bioinformatics 25, 1754–1760 (2009).** |
| **FigTree v1.4.2** | **http://tree.bio.ed.ac.uk/software/figtree/** |
| **GenomeAnalysisTK v3.7** | **McKenna, A. et al. The Genome Analysis Toolkit: a MapReduce framework for analyzing next-generation DNA sequencing data. Genome Res. 20, 1297–303 (2010).** |
| **IGV v2.3.68** | **https://software.broadinstitute.org/software/igv/** |
| **java v1.8.0_91** | **http://www.oracle.com/technetwork/java/index.html** |
| **Network v4.614** | **http://www.fluxus-engineering.com/sharenet.htm** |
| **picard-tools v2.3.0** | [**http://broadinstitute.github.io/picard**](http://broadinstitute.github.io/picard) |
| **Primer3** | [**http://bioinfo.ut.ee/primer3-0.4.0/**](http://bioinfo.ut.ee/primer3-0.4.0/) |
| **Python v3.5.1** | [**http://www.python.org**](http://www.python.org) |
| **R v3.2.3** | **R Core Team (2017). R: A language and environment for statistical computing. R Foundation for Statistical Computing, Vienna, Austria. URL http://www.R-project.org/** |
| **ReadTools v.0.2.1.r_716422a3** | **Gómez-Sánchez, D. & Schlötterer, C. ReadTools: A universal toolkit for handling sequence data from different sequencing platforms. Mol. Ecol. Resour. 18, 676–680 (2018).** |
| **samtools v1.4** | **Li, H. et al. The Sequence Alignment / Map format and SAMtools. Bioinformatics 25, 2078–2079 (2009).** |
| **seqtk v1.2-r94** | **seqtk, Toolkit for processing sequences in FASTA/Q formats. Available from:** [**https://github.com/lh3/seqtk**](https://github.com/lh3/seqtk)**.** |
| **SPAdes v3.11.1** | **Bankevich, A. et al. SPAdes: A New Genome Assembly Algorithm and Its Applications to Single-Cell Sequencing. J. Comput. Biol. 19, 455–477 (2012).** |
| **Sratoolkit v2.8.2** | [**https://www.ncbi.nlm.nih.gov/sra/docs/toolkitsoft/**](https://www.ncbi.nlm.nih.gov/sra/docs/toolkitsoft/) |

**Supplementary Figure legends**

**Supplementary Figure S1.** First step for the normalisation of the NGS data. For each contig the percentage of its length covered on average by males vs. females is shown. 100% Y-specific contigs cluster in the lower right corner of the plot (100% covered by males and 0% by females). The red line was put artificially to roughly separate putative Y-specific and not Y-specific contigs – this was necessary for step three of the normalisation, as explained in Supplementary Figure S3 and the Supplementary Methods.

**Supplementary Figure S2.** Second step for the normalisation of the NGS data. For each male the average depth of each contig clustering in the lower right corner of Supplementary Figure S1 (100% Y-specific contigs) was plotted – the plot for one male is shown as demonstration. The first peak of the distribution corresponds to single copy Y-specific contigs and its mode (8) was used to normalise the data of the sample shown such that a relative coverage of one corresponds to a haploid state.

**Supplementary Figure S3.** Third step for the normalisation of the NGS data. Knowing the average coverage of single copy Y-specific contigs for each male, it was possible to define putatively single copy autosomal contigs, which cluster above the red line shown in Supplementary Figure S1. For each female the average depth of these contigs was plotted – the plot for one female is shown as demonstration. The first peak of the distribution corresponds to single copy contigs and half of its mode (19/2=9.5) is used to normalise the data of the sample shown such that a relative coverage of one corresponds to a haploid state.

**Supplementary Figure S4.** Distribution of the normalised mapping coverages of windows classified as scY or mcY. The red line is the threshold (2) we used to separate scY (left) from mcY (right) and was chosen according to the distribution.

**Supplementary Figure S5.** The Y-specific content per contig is shown in percentage of its length. At least 50% of a contig‘s length have to be classified as scY or mcY for it to be kept as part of the final assembly CBacY1775.

**Supplementary Figure S6.** MSY haplotype network based on 152 scY SNVs found on the 329 CBacY1775 contigs used for dating (Supplementary Table S3). An artificial SNV OG was included to better visualize the root. Coloring and clustering correspond to Figure 2. Numbers indicate branch lengths (numbers of mutations). The rho estimates incl. confidence intervals for node ages are shown.

**Supplementary Figure S7.** A. Schematic overview of the nine tested SNVs. Orange variants have been successfully validated, for grey variants the validation failed, so far (see main text). The female sign highlights variants for which also female template DNA lead to positive PCR results as shown for cOO in part B. of the Figure. B. PCR products for four SNVs amplified from male (m; the first with the alternative, the second with the reference allele) and female (f) genomic DNA and a no-template-control. C. Electropherograms for four of the validated SNVs showing the SNV in the middle of each picture.
